# Value based competition in health care’s ethical drawbacks and the need for a values-driven approach

**DOI:** 10.1186/s12913-019-4081-6

**Published:** 2019-04-27

**Authors:** A. Stef Groenewoud, Gert P. Westert, Jan A. M. Kremer

**Affiliations:** Radboud University Medical Center, Scientific Center for Quality of Healthcare, PO Box 9101, 6500 HB Nijmegen, the Netherlands

**Keywords:** Value based health care, Ethics, Patient values, Values driven health care, Indicators, Trust, Accountability, Competition, Intrinsic value

## Abstract

Value Based competition in Health Care (VBHC) has become a guiding principle in the quest for high quality health care for acceptable costs. Current literature lacks substantial ethical evaluation of VBHC.

In this paper we describe how a single-minded focus on VBHC may cause serious infringements upon at least four medical ethical principles: 1) it tends to neglect patients’ personal values; 2) it ignores the intrinsic value of the caring act; 3) it disproportionately replaces trust in professionals with accountability, and 4) it undermines solidarity.

Health care needs a next step in VBHC. We suggest a ‘Values-Driven Health Care’ (VDHC) approach that a) takes patients’ personal values as prescriptive and guiding; b) holds a value account that encompasses health care’s intrinsic (gift) values; c) is based upon intelligent accountability that supports trust in trustworthy professionals, and d) encourages patients to raise their voices for the shared good of health care.

## Background

In 2006 the concept of Value Based competition in Health Care (VBHC) was introduced by Porter, professor of business strategy at Harvard Business School [1]. During the previous years, health care in the US, and other Western countries had been heavily criticized for its high costs and suboptimal quality and safety [2]. With VBHC Porter introduced a twofold strategy to tackle this. First, health care should be reorganized into *‘value-based’* care pathways around patient groups. These pathways should no longer be merely focused on increasing production, while shifting costs to other providers, but they should strive for the highest value for patients, i.e. the best possible outcomes, for acceptable costs [3]. Second, these pathways should *compete* for the favour of patients and health care purchasers. Patients should – in turn – behave more as critical health care consumers, while purchasers ‘buy’ the best possible health care for the lowest possible costs.

VBHC’s uptake is immense. During the last decade, VBHC has been embraced by almost all stakeholders in most Western health care systems. In the US for example, there is an extended hospital value-based purchasing program led by the Centers for Medicare & Medicaid Services (CMS) [4]. In the UK, the NHS officially made a shift toward VBHC in 2017, when the NHS Right Care program officially endorsed the approach in order to *“(...) drive value-based healthcare at a population level, improve patient outcomes and deliver significant cost savings”* [5]. Recently, ‘The Economist’ assigned Sweden as the worldwide leader in VBHC adoption [6].

Although it is widely believed that VBHC contributes to more efficient clinical pathways, a focus on relevant outcomes, cost awareness, and transparency, evidence of its effectiveness is still scarcely available [7, 8]. Besides, it is not self evident that VBHC’s concepts, that are mainly taken from business strategy, can be straightforwardly translated to the context of health care. For example, in 1963, health economist Kenneth Arrow already showed that (free) competition is not the most suitable distribution mechanism for health care [9]. During the nineties, Edmund Pellegrino discussed how the introduction of a business ethics into health care would corrode important health care values [10]. And, last but not least, the definition and the conception of ‘value’ is a completely different one in (health care) ethics, and philosophy compared to VBHC’s value definition: ‘outcomes divided by costs’ [11].

Given VBHC’s considerable influence, the lacking evidence, and the questions that may be raised regarding the suitability of a business strategy approach for health care, it is remarkable that – to our best knowledge – no substantial ethical reflection upon VBHC has been published yet. This article’s aim is to fill this gap by ethically reviewing the concept of VBHC. We show that a single minded focus on some of VBHC’s ideas may cause serious infringements upon at least four medical ethical principles. The structure of this article is as follows: for each of the four critiques we first give some conceptual grounding, followed by a short vignette that illustrates the problem. We then analyse the cause of each infringement, and show what can be done to overcome the problem. This results into a proposal for a more Values Driven approach.

### Main text

#### Conceptual basis for the first two critiques

The first two critiques are caused by a rather *narrow conception of ‘value’* in VBHC. Whereas VBHC conceives of value in a merely economic way (‘clinical outcomes divided by costs’ [3]), ‘Value Theory’ in philosophy holds a much broader definition. Its broadest conception of value covers all parts of moral philosophy, social and political philosophy, aesthetics, feminist philosophy and the philosophy of religion. Thus, it encompasses all areas of philosophy that have some ‘evaluative’ aspect. Economic value might be part of this, but ‘*moral value*’ is at least as important. In its narrowest sense, ‘value theory’ is used for a relatively narrow area of normative ethical theory, particularly of concern to consequentialists. Here, ‘value theory’ is roughly synonymous with ‘axiology’, which seeks to investigate what things are good, how good they are, and how their goodness is related to one another. In other words: “what stuffs are good: what is of value?” In the field of axiology two debates dominate: a) whether or not there is more than one fundamental value (the *monism/pluralism* debate), and b) whether things are only of *instrumental* value (because they help you to attain certain ‘higher’ goals) or whether there are things that are also of *intrinsic* value [11].

It is especially on these two questions where VBHC’s narrow value definition starts to cause problems. First, we will show that VBHC holds a rather monistic conception of value, because it perceives certain outcomes as dominant over others. Second, it will become clear that VBHC merely encompasses health care’s instrumental value, and does not take into account the *intrinsic* value of the caring act.

### VBHC’s monistic account of ‘value’ neglects patients’ pluralistic, personal values in life

Let us take a closer look at the case that is described in Table 1. While Ms. B’s personal values seem to have played only an implicit role, VBHC would have judged the treatment as ‘high value care’: her mood had become more tempered (improved outcome) and lithium is a relatively cheap medicine (acceptable costs). The problem here is that VBHC uses a monistic, and fixed outcome hierarchy, which is highly standardized for all patients with a certain disease [3]. For example, the ICHOM indicator standard Depression and Anxiety defines “symptoms of depression” as a ‘tier 1 indicator’, which should preferably be measured with the Patient Health Questionnaire (PHQ-9) [12]. Such a reductionist value account however, neglects the fact that patients’ personal values in life differ to an important extent. Traditionally ‘patient values’ are a patient’s unique preferences, concerns and expectations he or she brings to a clinical encounter. We believe the patients’ unique values in life should be prescriptive and action guiding; not standard sets of indicators. An example of a normative value theory in healthcare that explicitly takes into account patients’ unique values in life, is Values Based Medicine (VBM) [13]. While VBHC is purely outcome-driven with a focus on the (expected) clinical results relative to its costs, the focus of VBM is on the perspective of the particular patient in a given situation. Also, the broadly endorsed concept of Patient Centered Care (PCC) makes analogous objections to a monistic conception of patients’ values. PCC acknowledges the importance of respect for patients as unique living beings, and the obligation to know them as persons in context of their own social worlds, listen to them, inform them, and to respect them. PCC also stresses that a good outcome must be defined in terms of what is meaningful and valuable to the individual patient [14].

**Table 1:** VBHC’s monistic account of ‘value’ neglects patients’ pluralistic, personal values in life

*Ms. B, a 64-year old artist, was referred to a psychiatrist, Dr K. She had a history of occasional but increasingly disruptive episodes of hypomania. They decide to start on a course of lithium. Their decision was mainly based on clinical effectiveness, cost, and potential adverse side-effects respectively. Yet, after a few weeks, that decision turned out to have been wrong. That is, judged by her values as an artist. She explained that she did not really have a problem with the lithium, and that her mood had indeed been more stable. The problem she experienced was that she could no longer “see colors”. Colors had lost their emotional intensity, which, for her as an artist, was a disaster. Had her need to be able to ‘really see’ colors been more apparent at the time, then the evidence of lithium’s ‘emotional blunting’ effects would probably have been discussed at that stage. Ms. B. might still have decided to start on lithium but she would have been aware of the possibility of this side effect and her doctor would have been aware of the fact that this was a major concern to her.*	

Case based on: Fulford, K. W. M., 2008. Values-Based Practice: A New Partner to Evidence-Based Practice and A First for Psychiatry? Mens Sana Monographs 6(1):2,3

A good example of VBM’s, and PCC’s focus on personal values is the concept of Advance Care Planning (ACP), which enables “*individuals to define goals and preferences for future medical treatment and care, to discuss these goals and preferences with family and healthcare providers, and to record and review these preferences if appropriate”* [15]. In an ACP program in our hospital, we have recently seen how VBM’s principles, greatly enrich VBHC’s narrow value conception. We developed “Pal Pal”, a tablet-based tool for navigating elderly patients with cancer through the last phase of their lives [16]. The tool helps patients and their spouses to discover their goals in life and to clarify personal values. Pal Pal asks them, for example: “what would you preferably like to do on an average day?” It then addresses the question how healthcare could be arranged in such a way that it helps to accomplish these goals. For example; if a patient loves gardening, it is discussed how health care could enable him to continue gardening for as long as possible. Pal Pal also supports outcome measurement, using adaptive techniques in order to obtain personalized measures, depending on a patient’s personal values and goals. This may be a Patient Reported Outcome Measure (PROM), but sometimes other techniques (interviews or observations) are required.

### VBHC’s instrumental perception of health care neglects health care’s intrinsic ‘gift-value’

Which outcome would result from VBHC measuring the value (outcomes divided by costs) of the care both elderly men (in the case in Table 2) receive? Given all kinds of professional quality standards and the lower opportunity costs of professional care, the result might be that the professional home care is valued higher than the well-meant informal care that was given by daughter B, which probably does not meet the professional criteria and has higher opportunity costs. Although we all sympathize with the special act of caring by daughter B, VBHC does not seem to be able to take this into account. In our view, VBHC infringes upon, and possibly even corrodes health care’s intrinsic ‘*gift value’* [17]. VBHC holds a merely instrumental conception of health care value, believing that health care only adds value if it achieves good outcomes for acceptable costs. However, for some goods – and we believe this also applies to the ‘caring’ part of health care – value is at least partially constituted by the nonmarket motives for which they are given. They are valued as tokens of love, admiration, respect, honor, and so forth. In gift values the relationship between those who are involved in the transaction is relevant, as well as the altruistic character of the transaction, which is therefore of intrinsic value. This is in sharp contrast with the way competitive markets function. Here, transactions are unimportant in themselves, relations are impersonal ones, identities of participants are of no importance, and one is free to pursue one’s personal advantage unrestrained by any considerations for the possible disadvantage of others [18].

**Table 2:** VBHC’s instrumental perception of health care neglects health care’s intrinsic ‘gift-value’

In a quiet street in a small town, two women live next door to each other. Both have an elderly father suffering from dementia. Both neighbours have full-time, well-paid jobs. Unfortunately, their jobs are currently under pressure because of the care their fathers need. Woman A) hires 24/7 home care for her father, so she can keep on working, and he receives the care he needs. Woman B) decides to work part-time for three days, so she can take care of her father five days a week. Woman A) is proud as she tells her neighbour that the home care organization she hired, scores best in several rankings she found on the internet. Woman B) wonders how the care she gives to her own father would be valued in such a ranking. Moreover, as soon as she hears the price her neighbour pays for professional care, she feels as if her voluntary ‘gift’ to her father is trivialised and reduced to its cash equivalent.	

Even if VBHC would claim that they are only worried about the efficient allocation of scarce resources, and have nothing to say about the intrinsic (gift) value of health care, the problem still exists, because not paying attention to the intrinsic value of care giving would corrode that value, as became clear in the vignette, where woman B felt that her voluntary gift was reduced to the cash equivalent of the care she gives to her father.

The field of ‘Care Ethics’ does pay attention to the intrinsic value of the caring relation [19]. Care ethics recognizes that below the surface of outcome measurement, there are deeper layers that have to be captured in order to get a grip on health care quality. It starts with 1. the quality of recognition of a health need (*caring about*), followed by 2. the quality of *taking care of* (assuming some responsibility for the identified need and a response, consisting of both the physician’s delivery of technical care and an empathic or emotional engagement), 3. the quality of care giving (the direct action or actual physical work that is involved in the caring response), and 4. quality of care-receiving (the way the object of care will respond to the care it receives [20]. We suggest that a next step in VBHC would be to incorporate Care Ethics into its conception of ‘value’ in healthcare. Simply adding an extra performance indicator will not suffice here, as it requires a paradigm shift in VBHC’s conception of quality (measurement). Where VBHC uses the paradigm of “erklären” (to seek management-governed explanations of cause and effect), care ethics is about “verstehen” (the interpretative understanding of the meaning of human activities in a social context). In order to really understand a patient’s specific context, we suggest VBHC’s current focus on measuring indicators will be supplemented and enriched by other methods, such as interviews, (participative) observations, shadowing techniques, and narratives in order to get a taste of the deeper ‘layers’ of intrinsic value in healthcare. The urgency of this paradigm shift is more and more acknowledged. In the Netherlands for example, the Federation of Health Care Insurers (ZN) embraced narrative medicine as complementary to the normative measurement of health care quality for the purpose of value based health care purchasing [21].

### Conceptual foundation of the third and fourth critique

Our third and fourth critique on VBHC, have to do with VBHC’s focus on *competition* in health care. Doing so, VBHC combines two of the four basic models for the distribution of public services, that have been previously distinguished and described by LSE economist Julien Le Grand, namely: the ‘targets and performance management model’ (instead of the ‘trust-model’) and the ‘choice and competition model’ (instead of the ‘voice-model’) [22]. Le Grand’s four models represent different views on dealing with the question “what drives good results of public services and what helps improvement when results are bad?”

In the *trust model*, professionals (doctors, nurses and others) are simply trusted to know what is best for their users, and to deliver high-quality services without interference from government or any other source. Opponents of the trust model believe that on occasion, even knightly professionals can get it wrong. That some knights may even in reality be knaves, and that users might not always be satisfied with the service that they get. Hence, Le Grand suggests a second model: *the targets and performance management model*. This is the opposite of trust; a version of command and control where central management sets targets for providers, rewards them if they succeed in meeting those targets and penalizes them if they fail. In the *voice model* users express their dissatisfaction (or satisfaction) directly to providers through complaints to higher managers or elected representatives. In the *choice and competition model*, users choose between services offered by competing providers.

Our third critique has to do with the risk that VBHC may disproportionately replace trust in professionals with a culture of mistrust and accountability. The fourth critique discusses the disadvantages of preferring the exit-option over the strategy of ‘voice’ if patients would experience of suboptimal quality of care.

### VBHC disproportionately replaces trust in professionals with accountability

One of the expressions of ‘competition’ in VBHC is the mechanism of ‘targets and performance management’ [23]. This mechanism leads however to greater standardization, measurement, auditing and bureaucracy, as well as tighter organizational control over daily medical practice (which is illustrated by case 3 in Table 3). This ‘audit culture’ runs the risk of explicitly marginalising professionalism and professional standards. As a consequence, professional values are under pressure, changing professional ethics into business ethics. This could lead to patients being seen as purely profit or loss centres, and the patient-doctor relationship as subordinate to results [10]. Doctors experience stress, loss of ownership, and feel discouraged to develop new initiatives. We do not cherish a nostalgic and naïve desire to return to the early days of the medical profession, when people put their unquestioning trust in the knavish character of doctors [24]. We do however plea for ‘intelligent forms of accountability’, which may help to put trust in the trustworthy: the competent, the reliable and the honest [25]. Instead of measuring skills, and knowledge, the training of professionals will have to encompass ‘cultivation of virtues and character traits’. For instance in our hospital, the concept of ‘intelligent trust’ has led to proposals to change the existing competence profiles for medical students, which used to focus mainly on knowledge and skills. Our new medicine curriculum explicitly cultivates virtues as well as character traits, for example by using ‘role models’ for students and doctors, which has been claimed before to be an effective strategy [26, 27].

**Table 3:** VBHC disproportionately replaces trust in professionals with accountability

In a large hospital in London the board of directors organized a meeting with the Obstetrics and Gynaecology staff to discuss the newest set of outcome indicators for mother and child care. The implementation of the process of measuring, reporting, feedback, and improvement had not been a success thus far. As a result, the hospital had not been able to deliver its data to the NHS, who now gave them an ultimatum. One of the midwifes takes the floor, and she sounds rather agitated as she declares: *“Last week it took me longer to do the paperwork than to deliver the baby. That’s not what a midwife is there for”.*	

Based on: O’Neill, O., 2013. *What we don’t understand about trust. TED Talk:* https://www.ted.com/talks/onora_o_neill_what_we_don_t_understand_about_trust?language=nl . Accessed March 20, 2019

### VBHC may undermine solidarity by favouring exit over voice

According to VBHC “*patients and their families, (…) empowered with better information, would accept more responsibility for their health care choices. These choices have to be based on excellent results, not on convenience or amenities. The closest provider is not necessarily the best provider”* [28].

We think however that embracing the mechanism of choice as VBHC does, has three important disadvantages, all pointing towards an infringement upon the moral concept of ‘solidarity’. This infringement may gradually increase, depending on how one conceives of ‘solidarity’. In Anglo-Saxon countries, solidarity it is often used synonymously with the concept of ‘justice’ [29]. Here, solidarity means: equal freedom and equal opportunities for all persons in a society. Inequalities are acceptable as long as they work out for the good of the least well off. The *first* problem with VBHC’s idea of ‘voting with your feet’, based on information about health care quality, is that only well-educated people are able to do so (see also case 4 in Table 4). Lower educated people do not have equal opportunities, so to say, to end up in a high quality hospital. One could argue of course, that if all hospitals start improving their quality, the average quality will rise, and also the less well-off will be better off; which would indicate a fulfillment of the egalitarian criterion of justice [30]. However, to our knowledge there is no empirical prove for this expectation.

**Table 4:** VBHC may undermine solidarity by favouring exit over voice

Mrs. Z., a wealthy widow, visits an orthopaedist in her local, public hospital. On her first appointment at the outpatient ward, there appears to be ‘no love lost’ between her and her doctor, to put it mildly. Mrs. Z. leaves the hospital feeling very offended and insulted. That very evening she visits a website where patients can post reviews of their experiences with doctors. She writes a critical review, and she also uses the ‘find your doctor’ function to choose a different orthopaedist; this time in a private clinic. One week later, Mrs. Z. tells her story to her brother in law, Mr. J., who is not that well-established. He was not even aware of the fact that patients are ‘allowed’ to change doctors. Let alone that he knew the place on the internet where comparisons between health care providers can be made. He wonders what will happen if all people like Mrs. Z. will ‘leave the public system’. Deep in his heart, it makes him feel a second-rate civilian, who is left with inferior health care facilities.	

The second and third infringement of the moral concept of ‘solidarity’ become more clear if we follow the definition of solidarity, that was introduced by Prainsack and Buyx, conceiving of solidarity as: “*shared practices reflecting a collective commitment to carry ‘costs’ (financial, social, emotional,or otherwise) to assist others”* [31]. Encouraging patients to vote with their feet, as VBHC does, may incentivize (middle and higher classes) patients to escape the system, turning instead to private providers that are outside the reach of the less fortunate. Also, from a standpoint of the republican conception of citizenship, this is dangerous. This inequality does not simply prevent the poor from sharing in the fruits of consumption and choosing their ends for themselves; it also leads rich and poor to live increasingly separate lives [32].

Third, exit’ might even have degrading effects on health care’s *‘shared value’* [9]. This is the intrinsic value of those parts of society whose ‘being good’ consists – at least partly – in the fact that they are understood to be held in common. Shared values are dependent on people collectively enjoying them. We are all familiar with this from for example cultural heritage or National Parks, but why should we not cherish our health care systems in the same way? Shared enjoyment of health care also encompasses a shared responsibility for the improvement of the good, whenever a member of the group experiences problems or dissatisfaction in using it.

Where VBHC’s focus on choice and exit potentially degrades the value of healthcare, we suggest ‘voice’ as a completely different corrective, calling on people’s responsibility to defend the shared good of healthcare. ‘Voice’, in this sense, may come in many different forms. Most Western countries have institutionalized patient representation at both a meso level (e.g. hospital) and a macro level (e.g. benefit basket). Much more radical is the phenomenon of “community owned (primary) health care”, which is quite common in Australia, where civilians take responsibility to organize and improve health care, and (re)distribute the resources available [33].

## Conclusion

We have seen how VBHC may infringe upon at least four important values or principles in health care. VBHC’s rather narrow conception of value tends to neglect patients’ personal and contextual values of life, and it does not encompass the intrinsic value of the caring act. Besides, VBHC’s focus on target and performance managements (the business of outcome measurement), and on choice and exit (voting with your feet) may endanger the concepts of ‘trust in professionals’ respectively ‘solidarity’. Health care desperately needs a next step in VBHC. We believe this next step should be ‘Values-Driven Health Care’. In this article we have shown how this alternative approach would be of great complementary value to VBHC in the way it 1) takes patients’ personal values as prescriptive and guiding; 2) holds a value account that encompasses health care’s intrinsic (gift) values; 3) is based upon intelligent accountability that supports trust in the trustworthy, and 4) encourages patients to raise their voices for the shared good of health care.

## Abbreviations

ACP: Advance care planning
CMS: Centers for Medicare and Medicaid Services
PCC: Patient Centred Care
PROM: Patient Reported Outcome Measure
VBHC: Value based health care
VBM: Values based medicine
VDHC: Values Driven Health Care
